# Supplementary material for: Inferring synaptic transmission from the stochastic dynamics of the quantal content: An analytical approach
Source: PLoS Comput Biol. 2025 May 13;21(5):e1013067. doi: 10.1371/journal.pcbi.1013067 (PMC12101786; doi:10.1371/journal.pcbi.1013067)
Supplement: S1 File — (PDF) [file pcbi.1013067.s001.pdf]

Supporting information (S1 file) for:

Inferring synaptic transmission from the stochastic dynamics of the quantal content: An analytical approach

Appendix A

Let $k_{d,i}$ and $k_{u,i}$ be constant kinetic rates with which SVs dock and undock to each docking site, respectively, between the ith and $i+1^{th}$ AP. It is important to point out that these kinetic rates themselves depend on the rate of arrival of presynaptic APs. The probability $x(t)$ of an empty site to be occupied in the subsequent time t evolves as

$$\frac{dx}{dt} = k_{d,i}(1-x) - k_{u,i}x, \quad x(0) = 0. \tag{S1}$$

This results in the following refilling probability in time interval T_i between successive APs

$$p_{d,i} := x(T_i) = \frac{k_{d,i}}{k_{d,i}+k_{u,i}} \left(1 - e^{-(k_{d,i}+k_{u,i})T_i}\right). \tag{S2}$$

Similarly, solving (S1) with initial condition $x(0) = 1$ gives the probability of a docked SV undocking in time interval T_i

$$p_{u,i} = \frac{k_{u,i}}{k_{d,i}+k_{u,i}} \left(1 - e^{-(k_{d,i}+k_{u,i})T_i}\right). \tag{S3}$$

In the above formulation we assumed that replenishment at each empty site occurs as per a Poisson process with rate $k_{d,i}$. This can be generalized to an inhomogeneous Poisson process with SV docking and undocking at each site, occurring with rates $k_{d,i}(\tau)$ and $k_{u,i}(\tau)$ between the ith and $i+1^{th}$ AP, respectively. These rates are arbitrary functions of τ, and τ measures the time elapsed since the ith AP. Such rates can capture scenarios where a delay in the occurrence of the next AP also results in a lowering of the refilling rates as a result of depletion or buffering of calcium in the axon terminal. In this case refilling probability is obtained by solving

$$\frac{dx}{d\tau} = k_{d,i}(\tau)(1-x) - k_{u,i}(\tau)x, \quad x(0) = 0. \tag{S4}$$

that results in

$$p_{d,i} = e^{-\int_{y=0}^{T_i}(k_{d,i}(y)+k_{u,i}(y))dy} \int_{y=0}^{T_i} \left(k_{d,i}(y) e^{\int_{z=0}^{y}(k_{d,i}(z)+k_{u,i}(z))dz} dy\right). \tag{S5}$$

Similarly we can obtain

$$p_{u,i} = e^{-\int_{y=0}^{T_i}(k_{d,i}(y)+k_{u,i}(y))dy} \int_{y=0}^{T_i} \left(k_{u,i}(y) e^{\int_{z=0}^{y}(k_{d,i}(z)+k_{u,i}(z))dz} dy\right). \tag{S6}$$

Appendix B

A detailed proof can be found in the preprint [93] and is also provided here for convenience. We use the notation

$$\boldsymbol{n}_i \sim \mathcal{B}(M, p_i), \tag{S7}$$

to denote that the random variable $\boldsymbol{n}_i$ follows the binomial distribution with probability mass function (1) that is defined by M (the number of trials) and p_i (the success probability in each trial). The probability generating function (pgf) of $\boldsymbol{n}_i$ is given by

$$\langle z^{\boldsymbol{n}_i} \rangle = (1 - p_i + p_i z)^M . \tag{S8}$$

Conditioned on $\boldsymbol{n}_i$, the QC is also binomial $\boldsymbol{b}_i \sim \mathcal{B}(\boldsymbol{n}_i, p_{r,i})$, then $\boldsymbol{b}_i$ is itself binomially distributed with parameters

$$\boldsymbol{b}_i \sim \mathcal{B}(M, p_i p_{r,i}). \tag{S9}$$

To see this we first find

$$\left\langle z^{\boldsymbol{b}_i} | \boldsymbol{n}_i \right\rangle = (1 - p_{r,i} + p_{r,i} z)^{\boldsymbol{n}_i} , \tag{S10}$$

and then unconditioning with respect to $\boldsymbol{n}_i$

$$\begin{aligned} \left\langle z^{\boldsymbol{b}_i} \right\rangle &= \left\langle (1 - p_{r,i} + p_{r,i} z)^{\boldsymbol{n}_i} \right\rangle \\ &= (1 - p_i + p_i (1 - p_{r,i} + p_{r,i} z))^M \\ &= (1 - p_i p_{r,i} + p_i p_{r,i} z)^M \end{aligned} \tag{S11}$$

yields the pgf of $\boldsymbol{b}_i$ that is the same as that of a binomially distributed random variable with parameters M and $p_i p_{r,i}$.

To prove (S7), where p_i is the solution to (2), we use the method of induction, where we know (S7) is true for $i = 1$. Now assuming it to be true for any arbitrary stimulus number i we show it is also true for $i + 1$. Given $\boldsymbol{n}_i \sim \mathcal{B}(M, p_i)$, then the number of SVs $\boldsymbol{n}_i^+$ just after the ith AP will be

$$\boldsymbol{n}_i^+ \sim \mathcal{B}(M, p_i(1 - p_{r,i})). \tag{S12}$$

binomially distributed corresponding to each of the docked SVs not releasing with probability $1 - p_{r,i}$. The number of docked SVs just before the $i + 1^{th}$ AP is

$$\boldsymbol{n}_{i+1} = \boldsymbol{n}_i^+ - \boldsymbol{n}_{u,i} + \boldsymbol{n}_{d,i}. \tag{S13}$$

Here $\boldsymbol{n}_{u,i}$ is the number of docked SVs that undock between the ith and $i + 1^{th}$ AP, and conditioned on $\boldsymbol{n}_i^+$ it is given by

$$\boldsymbol{n}_{u,i} \sim \mathcal{B}(\boldsymbol{n}_i^+, p_{u,i}). \tag{S14}$$

Similarly, the number of empty sites that get occupied in the time interval between successive APs is

$$\boldsymbol{n}_{d,i} \sim \mathcal{B}(M - \boldsymbol{n}_i^+, p_{d,i}). \tag{S15}$$

Taking the pgf of $\boldsymbol{n}_{i+1}$ and using (S8)

$$\begin{aligned} \left\langle z^{\boldsymbol{n}_{i+1}} | \boldsymbol{n}_i^+ \right\rangle &= z^{\boldsymbol{n}_i^+} \left\langle z^{-\boldsymbol{n}_{u,i}} \right\rangle \left\langle z^{\boldsymbol{n}_{d,i}} \right\rangle \\ &= z^{\boldsymbol{n}_i^+} \left(1 - p_{u,i} + p_{u,i} z^{-1}\right)^{\boldsymbol{n}_i^+} (1 - p_{d,i} + p_{d,i} z)^{M - \boldsymbol{n}_i^+} \\ &= \left(z \frac{\left(1 - p_{u,i} + p_{u,i} z^{-1}\right)}{(1 - p_{d,i} + p_{d,i} z)} \right)^{\boldsymbol{n}_i^+} (1 - p_{d,i} + p_{d,i} z)^M . \end{aligned} \tag{S16}$$

Now unconditioning (S16) with respect to $\boldsymbol{n}_i^+$ and using (S12)

$$\begin{aligned}\left\langle z^{\boldsymbol{n}_{i+1}l}\right\rangle &= \left(1 - p_i(1-p_{r,i}) + p_i(1-p_{r,i})\frac{(p_{u,i} + (1-p_{u,i})z)}{(1-p_{d,i}+p_{d,i}z)}\right)^M (1-p_{d,i}+p_{d,i}z)^M \\ &= (1-p_{d,i}+p_{d,i}z - p_i(1-p_{r,i})\,(1-p_{d,i}+p_{d,i}z) + \\ &\quad p_i(1-p_{r,i})(p_{u,i}+(1-p_{u,i})z))^M \\ &= (1-p_{i+1}+p_{i+1}z)^M \end{aligned} \tag{S17}$$

where

$$p_{i+1} = p_i(1-p_{r,i})(1-p_{u,i}) + (1-p_i(1-p_{r,i}))\,p_{d,i}. \tag{S18}$$

The pgf (S17) shows that $\boldsymbol{n}_{i+1} \sim \mathcal{B}(M, p_{i+1})$ is a binomially-distributed random variable, hence completing the proof by induction.

Appendix C

If the time T_i between APs follows an independent and identically distributed (i.i.d.) random variable, then $p_{u,i}$ and $p_{d,i}$ are also i.i.d. random variables drawn as per (S2) and (S3) in Appendix A, and p_i is the solution to the random discrete-time system (2). In this case, conditioned on p_i, $\boldsymbol{b}_i$ is binomially distributed as per (3). However, since now p_i itself is a random variable, $\boldsymbol{b}_i$ will no longer be binomially distributed as has been reported in other works [51–53, 62]. For example in [53] we consider AP arrival as per a Poisson process where the inter-stimulus interval is an exponentially distributed random variable with mean $1/f$. Then using the standard tool of moment dynamics for Stochastic Hybrid Systems [94–96], the steady-state QC Fano factor FF is derived in [53, 97]. Interestingly, in this case, FF is maximized at an intermediate frequency f (see Fig 3 in [97]) and for many parameter regimes can be higher than one. Note that the Fano factor of a binomial-distributed random variable is always less than equal to one.

Appendix D

In this section, we provided an exact analytical solution for the QC autocorrelation function (ACF) that is mathematically defined as the steady-state Pearson correlation coefficient between $\boldsymbol{b}_i$ and $\boldsymbol{b}_{i+1}$ in limit $i \to \infty$.

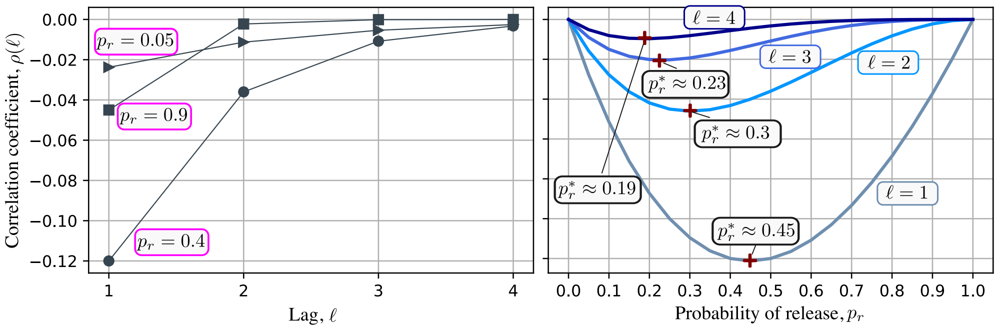

Fig A: The steady-state Pearson correlation coefficient between QCs $\boldsymbol{b}_i$ and $\boldsymbol{b}_{i+\ell}$ with lag $\ell \in \{1, 2, \ldots\}$ for different release probabilities with $p_d = 0.5$ (left). Correlation coefficient $\rho(\ell)$ as a function of release probability for different lags ℓ and $p_d = 0.4$ (right).

We first calculate the ACF at lag $\ell = 1$, conditioned on $\boldsymbol{b}_i$. Thus,

$$\langle \boldsymbol{b}_i \boldsymbol{b}_{i+1} | \boldsymbol{b}_i \rangle = \boldsymbol{b}_i \langle \boldsymbol{b}_{i+1} | \boldsymbol{b}_i \rangle. \tag{S19}$$

Given $\boldsymbol{n}_{i+1}$ and the AP-triggered binomial fusion of SVs with probability $p_{r,i+1}$, we can write

$$\text{Probability}\{\boldsymbol{b}_{i+1} = j | n_{i+1}\} = \binom{n_{i+1}}{j} (p_{r,i+1})^j \left(1 - p_{r,i+1}\right)^{n_{i+1}-j}, \quad j = \{0, \ldots, n_{i+1}\} \tag{S20}$$

and the mean QC given the number of docked vesicles at stimulus number $i+1$ follows

$$\langle \boldsymbol{b}_{i+1} | \boldsymbol{n}_{i+1} \rangle = \boldsymbol{n}_{i+1} p_{r,i+1}. \tag{S21}$$

Equation (S13) relates the number of docked SVs at $i+1^{th}$ AP to those at i^{th} AP. Thus, given $\boldsymbol{n}_i^+$, $\boldsymbol{n}_{u,i}$ and $\boldsymbol{n}_{d,i}$, $\boldsymbol{n}_{i+1}$ is known. Rewriting (S21)

$$\langle \boldsymbol{b}_{i+1} | \boldsymbol{n}_i^+, \boldsymbol{n}_{u,i}, \boldsymbol{n}_{d,i} \rangle = \left(\boldsymbol{n}_i^+ - \boldsymbol{n}_{u,i} + \boldsymbol{n}_{d,i}\right) p_{r,i+1}. \tag{S22}$$

Hence, by substituting (S22) in (S19) we obtain

$$\langle \boldsymbol{b}_i \boldsymbol{b}_{i+1} | \boldsymbol{n}_i^+, \boldsymbol{n}_{u,i}, \boldsymbol{n}_{d,i}, \boldsymbol{b}_i \rangle = \boldsymbol{b}_i \left(\boldsymbol{n}_i^+ - \boldsymbol{n}_{u,i} + \boldsymbol{n}_{d,i}\right) p_{r,i+1}. \tag{S23}$$

Substituting SVs after AP with those before minus QC yields

$$\boldsymbol{n}_i^+ = \boldsymbol{n}_i - \boldsymbol{b}_i, \tag{S24}$$

in (S23), we obtain

$$\langle \boldsymbol{b}_i \boldsymbol{b}_{i+1} | \boldsymbol{n}_i, \boldsymbol{n}_{u,i}, \boldsymbol{n}_{d,i}, \boldsymbol{b}_i \rangle = \boldsymbol{b}_i \left(\boldsymbol{n}_i - \boldsymbol{b}_i - \boldsymbol{n}_{u,i} + \boldsymbol{n}_{d,i}\right) p_{r,i+1}. \tag{S25}$$

And finally the above expression is simplified to

$$\langle \boldsymbol{b}_i \boldsymbol{b}_{i+1} \rangle = \left(\langle \boldsymbol{b}_i \boldsymbol{n}_i \rangle - \langle \boldsymbol{b}_i^2 \rangle - \langle \boldsymbol{b}_i \boldsymbol{n}_{u,i} \rangle + \langle \boldsymbol{b}_i \boldsymbol{n}_{d,i} \rangle\right) p_{r,i+1}. \tag{S26}$$

We next calculate $\langle \boldsymbol{b}_i \boldsymbol{n}_i \rangle$, $\langle \boldsymbol{b}_i^2 \rangle$, $\langle \boldsymbol{b}_i \boldsymbol{n}_{u,i} \rangle$, $\langle \boldsymbol{b}_i \boldsymbol{n}_{d,i} \rangle$.

Finding $\langle \boldsymbol{b}_i \boldsymbol{n}_i \rangle$:

$$\langle \boldsymbol{b}_i \boldsymbol{n}_i | \boldsymbol{n}_i \rangle = \langle \boldsymbol{b}_i | \boldsymbol{n}_i \rangle \boldsymbol{n}_i, \tag{S27}$$

and using (S20) for stimulus number i

$$\langle \boldsymbol{b}_i | \boldsymbol{n}_i \rangle = \boldsymbol{n}_i p_{r,i}. \tag{S28}$$

Also using the pmf (Probability Mass Function) in (S7),

$$\langle \boldsymbol{n}_i \rangle = M p_i \quad \text{and} \quad \langle \boldsymbol{n}_i^2 \rangle - \langle \boldsymbol{n}_i \rangle^2 = M p_i (1 - p_i) \quad \implies \quad \langle {\boldsymbol{n}_i}^2 \rangle = M p_i (1 - p_i) + M^2 p_i^2. \tag{S29}$$

Using (S27)-(S29)

$$\langle \boldsymbol{b}_i \boldsymbol{n}_i | \boldsymbol{n}_i \rangle = \boldsymbol{n}_i^2 p_{r,i} \quad \implies \quad \langle \boldsymbol{b}_i \boldsymbol{n}_i \rangle = \langle \boldsymbol{n}_i^2 \rangle p_{r,i} = \left(M p_i (1 - p_i) + M^2 p_i^2\right) p_{r,i}. \tag{S30}$$

Finding $\langle \boldsymbol{b}_i^2 \rangle$:

Using the pmf in (S9),

$$\begin{aligned} &\langle \boldsymbol{b}_i \rangle = M p_i p_{r,i} \quad \langle \boldsymbol{b}_i^2 \rangle - \langle \boldsymbol{b}_i \rangle^2 = M p_i p_{r,i} (1 - p_i p_{r,i}) \\ &\implies \langle \boldsymbol{b}_i^2 \rangle = M p_i p_{r,i} (1 - p_i p_{r,i}) + M^2 p_i^2 p_{r,i}^2. \end{aligned} \tag{S31}$$

Finding $\langle \boldsymbol{b}_i \boldsymbol{n}_{u,i} \rangle$:

Conditioned on $\boldsymbol{n}_i^+$ and $\boldsymbol{b}_i$

$$\langle \boldsymbol{b}_i \boldsymbol{n}_{u,i} | \boldsymbol{n}_i^+, \boldsymbol{b}_i \rangle = \boldsymbol{b}_i \langle \boldsymbol{n}_{u,i} | \boldsymbol{n}_i^+, \boldsymbol{b}_i \rangle. \tag{S32}$$

Using the pmf of $\boldsymbol{n}_{u,i}$ (S14)

$$\langle \boldsymbol{n}_{u,i} | \boldsymbol{n}_i^+, \boldsymbol{b}_i \rangle = \boldsymbol{n}_i^+ p_{u,i}, \tag{S33}$$

and from (S33) and (S24)

$$\langle \boldsymbol{n}_{u,i} | \boldsymbol{n}_i^+, \boldsymbol{b}_i \rangle = \langle \boldsymbol{n}_{u,i} | \boldsymbol{n}_i, \boldsymbol{b}_i \rangle = (\boldsymbol{n}_i - \boldsymbol{b}_i)\, p_{u,i}. \tag{S34}$$

Substituting (S34) in (S32) we obtain

$$\langle \boldsymbol{b}_i \boldsymbol{n}_{u,i} | \boldsymbol{n}_i, \boldsymbol{b}_i \rangle = \boldsymbol{b}_i \left(\boldsymbol{n}_i - \boldsymbol{b}_i\right) p_{u,i}. \tag{S35}$$

Next, we uncondition the above equation

$$\langle \boldsymbol{b}_i \boldsymbol{n}_{u,i} \rangle = \left(\langle \boldsymbol{b}_i \boldsymbol{n}_i \rangle - \langle \boldsymbol{b}_i^2 \rangle\right) p_{u,i}, \tag{S36}$$

with $\langle \boldsymbol{b}_i \boldsymbol{n}_i \rangle$ is in (S30) and $\langle \boldsymbol{b}_i^2 \rangle$ in (S31).

Finding $\langle \boldsymbol{b}_i \boldsymbol{n}_{d,i} \rangle$:

Given $\boldsymbol{n}_i$ and $\boldsymbol{b}_i$

$$\langle \boldsymbol{b}_i \boldsymbol{n}_{d,i} | \boldsymbol{n}_i, \boldsymbol{b}_i \rangle = \boldsymbol{b}_i \langle \boldsymbol{n}_{d,i} | \boldsymbol{n}_i \rangle. \tag{S37}$$

Using (S24) and the pmf of $\boldsymbol{n}_{d,i}$ in (S15) we can write

$$\langle \boldsymbol{n}_{d,i} | \boldsymbol{n}_i, \boldsymbol{b}_i \rangle = \left(M - \boldsymbol{n}_i^+\right) p_{d,i} = \left(M - \boldsymbol{n}_i + \boldsymbol{b}_i\right) p_{d,i} \tag{S38}$$

Then we substitute (S38) in (S37)

$$\langle \boldsymbol{b}_i \boldsymbol{n}_{d,i} | \boldsymbol{n}_i b_i \rangle = \boldsymbol{b}_i \left(M - \boldsymbol{n}_i + \boldsymbol{b}_i\right) p_{d,i} \quad \Longrightarrow \quad \langle \boldsymbol{b}_i \boldsymbol{n}_{d,i} \rangle = \left(M \langle \boldsymbol{b}_i \rangle - \langle \boldsymbol{b}_i \boldsymbol{n}_i \rangle + \langle \boldsymbol{b}_i^2 \rangle\right) p_{d,i}, \tag{S39}$$

with $\langle \boldsymbol{b}_i \rangle$ and $\langle \boldsymbol{b}_i^2 \rangle$ in (S31), and $\langle \boldsymbol{b}_i \boldsymbol{n}_i \rangle$ in (S30). Finally, we simplify (S26) by substituting $\langle \boldsymbol{b}_i \boldsymbol{n}_{u,i} \rangle$ from (S36) and $\langle \boldsymbol{b}_i \boldsymbol{n}_{d,i} \rangle$ from (S39)

$$\begin{aligned} \langle \boldsymbol{b}_i \boldsymbol{b}_{i+1} \rangle &= \left(\langle \boldsymbol{b}_i \boldsymbol{n}_i \rangle - \langle \boldsymbol{b}_i^2 \rangle - \langle \boldsymbol{b}_i \boldsymbol{n}_{u,i} \rangle + \langle \boldsymbol{b}_i \boldsymbol{n}_{d,i} \rangle\right) p_{r,i+1} \\ &= \left(\left(\langle \boldsymbol{b}_i \boldsymbol{n}_i \rangle - \langle \boldsymbol{b}_i^2 \rangle\right)\left(1 - p_{u,i} - p_{d,i}\right) + M \langle \boldsymbol{b}_i \rangle p_{d,i}\right) p_{r,i+1}. \end{aligned} \tag{S40}$$

Substituting $\langle \boldsymbol{b}_i \rangle$ and $\langle \boldsymbol{b}_i^2 \rangle$ from (S31), and $\langle \boldsymbol{b}_i \boldsymbol{n}_i \rangle$ from (S30) in (S40) we obtain the ACF as follows

$$\sum_i \boldsymbol{b}_{i+1} \boldsymbol{b}_i = \langle \boldsymbol{b}_{i+1} \boldsymbol{b}_i \rangle \langle \boldsymbol{b}_i \boldsymbol{b}_{i+1} \rangle \tag{S41}$$

$$= M p_i \left(M p_{d,i} + (M-1) p_i (1 - p_{d,i} - p_{u,i})(1 - p_{r,i})\right) p_{r,i} p_{r,i+1}, \tag{S42}$$

Next we compute the Pearson correlation coefficient for lag $\ell = 1$ as (20) with $\langle \boldsymbol{b}_i \rangle$ and $\langle \boldsymbol{b}_i^2 \rangle$ are the mean and second-order moments of QC, respectively. Using constant values of probabilities in (9), (S40) simplifies to

$$\langle \boldsymbol{b}_i \boldsymbol{b}_{i+1} \rangle = \left(\langle \boldsymbol{b}_i \boldsymbol{n}_i \rangle - \langle \boldsymbol{b}_i^2 \rangle\right)(1 - p_u - p_d) p_r + M \langle b_i \rangle p_d p_r. \tag{S43}$$

At the steady state

$$\lim_{i\to\infty}\langle \boldsymbol{b}_i\boldsymbol{b}_{i+1}\rangle = \left(\lim_{i\to\infty}\langle \boldsymbol{b}_i n_i\rangle - \langle \boldsymbol{b}_i^2\rangle\right)(1-p_u-p_d)p_r + Mp_dp_r\lim_{i\to\infty}\langle \boldsymbol{b}_i\rangle. \tag{S44}$$

We simplify (S30) and (S31) for constant values of probabilities and at the steady-state

$$\lim_{i\to\infty}\langle \boldsymbol{b}_i\boldsymbol{n}_i\rangle = \left(M\bar{p}(1-\bar{p}) + M^2\bar{p}^2\right)p_r, \tag{S45}$$

where $\bar{p}$ is the steady-state value of p_i (13). Also,

$$\lim_{i\to\infty}\langle \boldsymbol{b}_i\rangle = M\bar{p}p_r \quad \text{and} \quad \lim_{i\to\infty}\langle \boldsymbol{b}_i^2\rangle = M\bar{p}p_r(1-\bar{p}p_r) + M^2\bar{p}^2p_r^2, \tag{S46}$$

We substitute (12) in (S45) and (S46) and use them in (S44) to obtain $\langle \boldsymbol{b}_i\boldsymbol{b}_{i+1}\rangle$ at the steady-state as

$$\lim_{i\to\infty}\langle \boldsymbol{b}_i\boldsymbol{b}_{i+1}\rangle = \frac{M{p_d}^2{p_r}^2\left(M-(1-p_r)(1-p_d-p_u)\right)}{\left((p_d+p_u)(1-p_r)+p_r\right)^2}. \tag{S47}$$

The Pearson correlation coefficient (20) at the steady-state follows

$$\rho := \lim_{i\to\infty}\frac{\langle \boldsymbol{b}_{i+1}\boldsymbol{b}_i\rangle - \langle \boldsymbol{b}_i\rangle^2}{\langle \boldsymbol{b}_i^2\rangle - \langle \boldsymbol{b}_i\rangle^2} = -\frac{p_d(1-p_r)p_r(1-p_d-p_u)}{-2p_dp_r - p_rp_u + p_r + p_d + p_u} \tag{S48}$$

Next, we generalize our approach to calculate the exact analytical solution for the QC autocorrelation function (ACF) and the steady-state Pearson correlation coefficient between $\boldsymbol{b}_i$ and $\boldsymbol{b}_{i+\ell}$, where $\ell \in \{1,2,\ldots\}$. Assuming constant valued probabilities p_r, p_d and p_u, the ACF at lag $\ell = 1$ is calculated in (S43). Here we apply the same approach for $\ell = 2$ following the steps as in (S19)-(S43). Given $\boldsymbol{b}_i$,

$$\langle \boldsymbol{b}_i\boldsymbol{b}_{i+2}|\boldsymbol{b}_i\rangle = \boldsymbol{b}_i\langle \boldsymbol{b}_{i+2}|\boldsymbol{b}_i\rangle, \tag{S49}$$

and give $\boldsymbol{n}_{i+2}$

$$\langle \boldsymbol{b}_{i+2}|\boldsymbol{n}_{i+2}\rangle = \boldsymbol{n}_{i+2}p_r. \tag{S50}$$

Similar to (S13)

$$\boldsymbol{n}_{i+2} = \boldsymbol{n}_{i+1}^{+} - \boldsymbol{n}_{u,i+1} + \boldsymbol{n}_{d,i+1}, \tag{S51}$$

and similar to (S24)

$$\boldsymbol{n}_{i+1}^{+} = \boldsymbol{n}_{i+1} - \boldsymbol{b}_{i+1}. \tag{S52}$$

Using (S50)-(S52) in (S49)

$$\langle \boldsymbol{b}_i\boldsymbol{b}_{i+2}|\boldsymbol{b}_i, \boldsymbol{n}_{i+1}, \boldsymbol{b}_{i+1}, \boldsymbol{n}_{u,i+1}, \boldsymbol{n}_{d,i+1}\rangle = \boldsymbol{b}_i\left(\boldsymbol{n}_{i+1} - \boldsymbol{b}_{i+1} - \boldsymbol{n}_{u,i+1} + \boldsymbol{n}_{d,i+1}\right)p_r \tag{S53}$$

and by unconditioning it we obtain

$$\langle \boldsymbol{b}_i\boldsymbol{b}_{i+2}\rangle = \left(\langle \boldsymbol{b}_i\boldsymbol{n}_{i+1}\rangle - \langle \boldsymbol{b}_i\boldsymbol{b}_{i+1}\rangle - \langle \boldsymbol{b}_i\boldsymbol{n}_{u,i+1}\rangle + \langle \boldsymbol{b}_i\boldsymbol{n}_{d,i+1}\rangle\right)p_r. \tag{S54}$$

To compute $\langle \boldsymbol{b}_i\boldsymbol{b}_{i+2}\rangle$ we need $\langle \boldsymbol{b}_i\boldsymbol{n}_{i+1}\rangle$, $\langle \boldsymbol{b}_i\boldsymbol{n}_{u,i+1}\rangle$ and $\langle \boldsymbol{b}_i\boldsymbol{n}_{d,i+1}\rangle$ which we will find next.

Finding $\langle \boldsymbol{b}_i\boldsymbol{n}_{i+1}\rangle$:

$$\langle \boldsymbol{b}_i\boldsymbol{b}_{i+1}|\boldsymbol{b}_i, \boldsymbol{n}_{i+1}\rangle = \boldsymbol{b}_i\langle \boldsymbol{b}_{i+1}|\boldsymbol{n}_{i+1}\rangle = \boldsymbol{b}_i\boldsymbol{n}_{i+1}p_r \quad \Longrightarrow \quad \langle \boldsymbol{b}_i\boldsymbol{n}_{i+1}\rangle = \langle \boldsymbol{b}_i\boldsymbol{b}_{i+1}\rangle/p_r. \tag{S55}$$

Finding $\langle \boldsymbol{b}_i \boldsymbol{n}_{u,i+1} \rangle$:

$$\langle \boldsymbol{b}_i \boldsymbol{n}_{u,i+1} | \boldsymbol{b}_i \rangle = \boldsymbol{b}_i \langle \boldsymbol{n}_{u,i+1} | \boldsymbol{b}_i \rangle, \tag{S56}$$

and similar to (S34)

$$\langle \boldsymbol{n}_{u,i+1} | \boldsymbol{n}^+_{i+1}, \boldsymbol{b}_{i+1} \rangle = \langle \boldsymbol{n}_{u,i+1} | \boldsymbol{n}_{i+1}, \boldsymbol{b}_{i+1} \rangle = (\boldsymbol{n}_{i+1} - \boldsymbol{b}_{i+1})\, p_u. \tag{S57}$$

Using (S56) and (S57)

$$\langle \boldsymbol{b}_i \boldsymbol{n}_{u,i+1} | \boldsymbol{b}_i, \boldsymbol{n}_{i+1}, \boldsymbol{b}_{i+1} \rangle = (\boldsymbol{b}_i \boldsymbol{n}_{i+1} - \boldsymbol{b}_i \boldsymbol{b}_{i+1})\, p_u \implies \langle \boldsymbol{b}_i \boldsymbol{n}_{u,i+1} \rangle = (\langle \boldsymbol{b}_i \boldsymbol{n}_{i+1} \rangle - \langle \boldsymbol{b}_i \boldsymbol{b}_{i+1} \rangle)\, p_u. \tag{S58}$$

Next, using (S55), we simplify the above equation

$$\langle \boldsymbol{b}_i \boldsymbol{n}_{u,i+1} \rangle = (\langle \boldsymbol{b}_i \boldsymbol{b}_{i+1} \rangle / p_r - \langle \boldsymbol{b}_i \boldsymbol{b}_{i+1} \rangle)\, p_u = \langle \boldsymbol{b}_i \boldsymbol{b}_{i+1} \rangle (1/p_r - 1) p_u. \tag{S59}$$

Finding $\langle \boldsymbol{b}_i \boldsymbol{n}_{d,i+1} \rangle$:

$$\langle \boldsymbol{b}_i \boldsymbol{n}_{d,i+1} | \boldsymbol{b}_i \rangle = \boldsymbol{b}_i \langle \boldsymbol{n}_{d,i+1} | \boldsymbol{b}_i \rangle, \tag{S60}$$

and similar to (S38)

$$\langle \boldsymbol{n}_{d,i+1} | \boldsymbol{n}_{i+1}, \boldsymbol{b}_{i+1} \rangle = \left(M - \boldsymbol{n}^+_{i+1}\right) p_d = (M - \boldsymbol{n}_{i+1} + \boldsymbol{b}_{i+1})\, p_d. \tag{S61}$$

Using (S60) and (S61) we write

$$\begin{aligned} &\langle \boldsymbol{b}_i \boldsymbol{n}_{d,i+1} | \boldsymbol{b}_i, \boldsymbol{n}_{i+1}, \boldsymbol{b}_{i+1} \rangle = (\boldsymbol{b}_i M - \boldsymbol{b}_i \boldsymbol{n}_{i+1} + \boldsymbol{b}_i \boldsymbol{b}_{i+1})\, p_d \\ &\implies \langle \boldsymbol{b}_i \boldsymbol{n}_{d,i+1} \rangle = (M \langle \boldsymbol{b}_i \rangle - \langle \boldsymbol{b}_i \boldsymbol{n}_{i+1} \rangle + \langle \boldsymbol{b}_i \boldsymbol{b}_{i+1} \rangle)\, p_d. \end{aligned} \tag{S62}$$

Applying (S55) in (S62)

$$\langle \boldsymbol{b}_i \boldsymbol{n}_{d,i+1} \rangle = (M \langle \boldsymbol{b}_i \rangle - \langle \boldsymbol{b}_i \boldsymbol{b}_{i+1} \rangle / p_r + \langle \boldsymbol{b}_i \boldsymbol{b}_{i+1} \rangle)\, p_d. \tag{S63}$$

Replacing (S55), (S59) and (S63) in (S54) we obtain

$$\langle \boldsymbol{b}_i \boldsymbol{b}_{i+2} \rangle = \langle \boldsymbol{b}_i \boldsymbol{b}_{i+1} \rangle (1 - p_r)(1 - p_d - p_u) + M p_d p_r \langle \boldsymbol{b}_i \rangle. \tag{S64}$$

Using (S43) in the above equation follows

$$\langle \boldsymbol{b}_i \boldsymbol{b}_{i+2} \rangle = \left(\left(\langle \boldsymbol{b}_i \boldsymbol{n}_i \rangle - \langle \boldsymbol{b}_i^2 \rangle \right) (1 - p_u - p_d) p_r + M \langle \boldsymbol{b}_i \rangle p_d p_r \right) (1 - p_r)(1 - p_d - p_u) + M p_d p_r \langle \boldsymbol{b}_i \rangle, \tag{S65}$$

and at the steady-state

$$\begin{aligned} \lim_{i\to\infty} \langle \boldsymbol{b}_i \boldsymbol{b}_{i+2} \rangle = &\left(\left(\lim_{i\to\infty} \langle \boldsymbol{b}_i n_i \rangle - \lim_{i\to\infty} \langle \boldsymbol{b}_i^2 \rangle \right) (1 - p_u - p_d) p_r + M p_d p_r \lim_{i\to\infty} \langle \boldsymbol{b}_i \rangle \right) \\ &\times (1 - p_r)(1 - p_d - p_u) + M p_d p_r \lim_{i\to\infty} \langle \boldsymbol{b}_i \rangle. \end{aligned} \tag{S66}$$

We use (S45) and (S46) in the above equation when $\bar{p}$ follows (12). Thus,

$$\lim_{i\to\infty} \langle \boldsymbol{b}_i \boldsymbol{b}_{i+2} \rangle = \frac{M {p_d}^2 {p_r}^2 \left(M - (1 - p_r)^2 (1 - p_d - p_u)^2 \right)}{\left((p_d + p_u)(1 - p_r) + p_r \right)^2}. \tag{S67}$$

Following the same steps for $\ell = 2$ in (S49)-(S67), and by induction we obtain the following expression when

$$\langle \boldsymbol{b}_i \boldsymbol{b}_{i+\ell} \rangle = \langle \boldsymbol{b}_i \boldsymbol{b}_{i+\ell-1} \rangle (1 - p_r)(1 - p_d - p_u) + \langle \boldsymbol{b}_i \rangle M p_d p_r, \quad \ell > 1 \tag{S68}$$

and its steady-state follows

$$\lim_{i\to\infty} \langle \boldsymbol{b}_i \boldsymbol{b}_{i+\ell} \rangle = \frac{M{p_d}^2{p_r}^2\left(M-(1-p_r)^\ell(1-p_d-p_u)^\ell\right)}{\left((p_d+p_u)(1-p_r)+p_r\right)^2}, \quad \ell \geq 1, \tag{S69}$$

Finally, the steady-state Pearson correlation coefficient follows

$$\rho(\ell) := \lim_{i\to\infty} \frac{\langle \boldsymbol{b}_{i+\ell}\boldsymbol{b}_i\rangle - \langle \boldsymbol{b}_i\rangle^2}{\langle \boldsymbol{b}_i^2\rangle - \langle \boldsymbol{b}_i\rangle^2} = -\frac{p_d p_r(1-p_r)^\ell(1-p_d-p_u)^\ell}{p_r+p_d+p_u-2p_d p_r - p_r p_u}, \quad \ell \geq 1.$$

Plots of $\rho(\ell)$ as a function of lag ℓ and p_r are shown in Fig A in Appendix D.

Appendix E

To quantify the effects of stochastic variations in quantal size we consider the PSC amplitude elicited upon the fusion of a single SV to be a random variable $c_{i,j}$ corresponding to the jth SV released in the ith stimulus. Then, the amplitude of the PSC that is evoked by the ith stimulus given $\boldsymbol{b}_i$ vesicles were released, is

$$\boldsymbol{e}_i = \sum_{j=1}^{\boldsymbol{b}_i} \boldsymbol{c}_{i,j}. \tag{S70}$$

We assume that all $\boldsymbol{c}_{i,j}$ are independent and identically distributed random variables with mean $\langle \boldsymbol{c}\rangle$ and coefficient of variation CV_q. Both $\langle \boldsymbol{c}\rangle$ and CV_q could be time-varying reflecting changes in quantal size during an AP train. Then,

$$\langle \boldsymbol{e}_i\rangle = \langle \boldsymbol{c}\rangle\langle \boldsymbol{b}_i\rangle, \tag{S71}$$

$$\langle \boldsymbol{e}_i^2\rangle = \langle \boldsymbol{b}_i\rangle\langle \boldsymbol{c}\rangle^2\left(1+CV_q^2\right) + \langle \boldsymbol{b}_i(\boldsymbol{b}_i-1)\rangle\langle \boldsymbol{c}\rangle^2 = \langle \boldsymbol{b}_i\rangle\langle \boldsymbol{c}\rangle^2 CV_q^2 + \langle \boldsymbol{c}\rangle^2\langle \boldsymbol{b}_i^2\rangle, \tag{S72}$$

$$\langle \boldsymbol{e}_i^2\rangle - \langle \boldsymbol{e}_i\rangle^2 = \langle \boldsymbol{b}_i\rangle\langle \boldsymbol{c}\rangle^2 CV_q^2 + \langle \boldsymbol{c}\rangle^2\left(\langle \boldsymbol{b}_i^2\rangle - \langle \boldsymbol{b}_i\rangle^2\right), \tag{S73}$$

and the Fano factor of $\boldsymbol{e}_i$ is given by

$$FF_i^e := \frac{\langle \boldsymbol{e}_i^2\rangle - \langle \boldsymbol{e}_i\rangle^2}{\langle \boldsymbol{e}_i\rangle} = \frac{\langle \boldsymbol{b}_i\rangle\langle \boldsymbol{c}\rangle^2 CV_q^2 + \langle \boldsymbol{c}\rangle^2\left(\langle \boldsymbol{b}_i^2\rangle - \langle \boldsymbol{b}_i\rangle^2\right)}{\langle \boldsymbol{c}\rangle\langle \boldsymbol{b}_i\rangle} \tag{S74}$$

$$= \langle \boldsymbol{c}\rangle FF_i + \langle \boldsymbol{c}\rangle CV_q^2, \tag{S75}$$

where FF_i is the Fano factor of QC defined in (6). Assuming all statistical moments converge to their respective steady-state values as $i \to \infty$, the steady-state Fano factor is obtained as

$$FF^e := \lim_{i\to\infty} FF_i^e = \langle \boldsymbol{c}\rangle FF + \langle \boldsymbol{c}\rangle CV_q^2$$

where $\langle \boldsymbol{b}\rangle := \lim_{i\to\infty}\langle \boldsymbol{b}_i\rangle$ and $FF := \lim_{i\to\infty} FF_i$. As expected, for a fixed quantal size $CV_q^2 = 0$, $FF^e = \langle \boldsymbol{c}\rangle FF$.

We next turn our attention to the steady-state Pearson correlation coefficient between successive evoked PSCs

$$\rho^e := \lim_{i\to\infty} \frac{\langle \boldsymbol{e}_{i+1}\boldsymbol{e}_i\rangle - \langle \boldsymbol{e}_i\rangle^2}{\langle \boldsymbol{e}_i^2\rangle - \langle \boldsymbol{e}_i\rangle^2}. \tag{S76}$$

From (S70) one can see that

$$\langle \boldsymbol{e}_{i+1}\boldsymbol{e}_i\rangle = \langle \boldsymbol{b}_{i+1}\boldsymbol{b}_i\rangle\langle \boldsymbol{c}\rangle^2, \tag{S77}$$

which using (S71)-(S73) yields

$$\rho^e := \lim_{i\to\infty} \frac{\langle \boldsymbol{e}_{i+1}\boldsymbol{e}_i\rangle - \langle \boldsymbol{e}_i\rangle^2}{\langle \boldsymbol{e}_i^2\rangle - \langle \boldsymbol{e}_i\rangle^2} = \lim_{i\to\infty} \frac{\langle \boldsymbol{b}_{i+1}\boldsymbol{b}_i\rangle\langle \boldsymbol{c}\rangle^2 - \langle \boldsymbol{b}_i\rangle^2\langle \boldsymbol{c}\rangle^2}{\langle \boldsymbol{b}_i\rangle\langle \boldsymbol{c}\rangle^2 CV_q^2 + \langle \boldsymbol{c}\rangle^2\left(\langle \boldsymbol{b}_i^2\rangle - \langle \boldsymbol{b}_i\rangle^2\right)} \tag{S78}$$

$$= \lim_{i\to\infty} \frac{\langle \boldsymbol{b}_{i+1}\boldsymbol{b}_i\rangle - \langle \boldsymbol{b}_i\rangle^2}{\langle \boldsymbol{b}_i\rangle CV_q^2 + \langle \boldsymbol{b}_i^2\rangle - \langle \boldsymbol{b}_i\rangle^2}. \tag{S79}$$

Rewriting (S79) in terms of ρ, the steady-state Pearson correlation coefficient between successive QCs defined as

$$\rho := \lim_{i\to\infty} \frac{\langle \boldsymbol{b}_{i+1}\boldsymbol{b}_i\rangle - \langle \boldsymbol{b}_i\rangle^2}{\langle \boldsymbol{b}_i^2\rangle - \langle \boldsymbol{b}_i\rangle^2}, \tag{S80}$$

yields

$$\rho^e = \lim_{i\to\infty} \frac{\langle \boldsymbol{b}_{i+1}\boldsymbol{b}_i\rangle - \langle \boldsymbol{b}_i\rangle^2}{\langle \boldsymbol{b}_i\rangle CV_q^2 + \langle \boldsymbol{b}_i^2\rangle - \langle \boldsymbol{b}_i\rangle^2} = \frac{\rho}{1 + \frac{CV_q^2}{FF}}, \tag{S81}$$

where FF is the steady-state Fano factor of $\boldsymbol{b}_i$.

Recall from Fig 5, that our experimental observations of steady-state statistics of QC (as obtained by dividing the evoked PSCs with the mean quantal size) show

$$\frac{FF^e}{\langle \boldsymbol{c}\rangle} = CV_q^2 + FF \approx 0.5 \tag{S82}$$

$$\rho^e = \frac{\rho}{1 + \frac{CV_q^2}{FF}} \approx -0.035. \tag{S83}$$

In the main text, we solved these two equations simultaneously for $CV_q^2 = 0$ with FF and ρ given by equations (16) and (20), respectively, for p_r and p_d. To study the impact of quantal size fluctuations, we solve these equations numerically for increasing values of CV_q^2. The results are shown in Fig B in Appendix E where with increasing CV_q, p_r shows a slight decrease (from 0.93 to 0.91) and p_d shows an increase (from 0.53 to 0.70) as CV_q increases from 0 to 40%. Quantal size data from individual MNTB-LSO synaptic connections reveal fluctuations of magnitude $CV_q \approx 10 - 20\%$. Based on our results in Fig B, we don't expect significant changes in the inferred parameter values in this range. Note from (S82) that the experimentally measured $FF^e/\langle \boldsymbol{c}\rangle \approx 0.5$ creates an upper bound on high CV_q can be before FF in (S82) becomes negative and a solution to these equations ceases to exist.

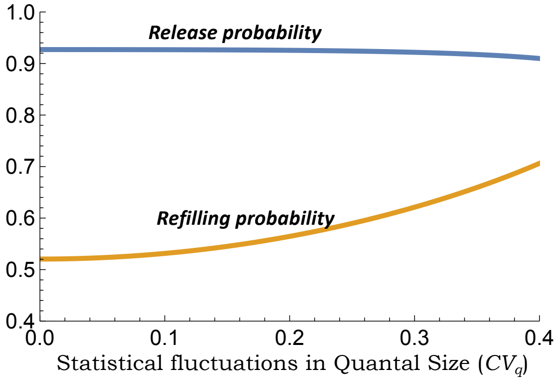

Fig B: The effect of quantal size fluctuations on inferred release and refilling probabilities as obtained by solving (S82)-(S83) with increasing values of CV_q, the coefficient of variation in quantal size (x-axis).

Appendix F

In this section we provide a maximum likelihood approach for characterizing the transient changes in the refilling probabilities that explicitly takes into account statistical fluctuations in quantal size. The parameters to be inferred consists of

$$\theta = [p_{d,1}, p_{d,2}, p_{d,3}, p_{d,4}, p_{d,5}, p_d] \tag{S84}$$

that correspond to the different refilling probabilities $p_{d,i}$ for the first five stimuli, and then a uniform refilling probability $p_d = p_{d,i}$ for the remaining stimuli ($\forall i \geq 6$). To simplify the optimization procedure we fix certain parameters values as obtained from the steady-state analysis of QC statistics in Fig 6. The steady-state analysis of QC data from 2900 stimuli (stimulus number 100 to 3000 in Fig 5A) revealed a high probability of release $p_r = 0.93$ (Fig 6C). For the likelihood estimation we fix this value of p_r and also the number of docking sites $M = 29$ that are all initially occupied by SVs to match it with the data showing a release of $29 * 0.93 \approx 27$ vesicles for the first stimulus.

Given the stimulus-dependent refilling probability (S84), our analytical results provide an exact transient statistical distribution of QC. For the ith AP, the QC is a binomial random variable

$$\text{Probability}\{\boldsymbol{b}_i = j\} = \binom{M}{j} (p_i p_r)^j \left(1 - p_i p_r\right)^{M-j}, \quad j = \{0, \ldots, M\}, \tag{S85}$$

where p_i is the solution to (2) and depends on the parameter set θ.

We next factor in quantal size fluctuations to predict the evoked PSC in response to a QC of j vesicles. As has been modeled before [98], we let quantal sizes $c_{i,j}$ in (S70) be independent and identically distributed random variables that follow a gamma distribution with probability density function (pdf) $f(x, \langle c \rangle, CV_q)$ with mean quantal size $\langle c \rangle$ and coefficient of variation CV_q. Since a sum of independent gamma-distributed random variables in (S70) is also gamma-distributed, given a QC of j vesicles, the evoked PSC response will be a gamma-distributed random variable with mean $\langle c \rangle j$ and coefficient of variation $CV_q/\sqrt{j}$, and have a pdf $f(x, \langle c \rangle j, CV_q/\sqrt{j})$. Thus, the probability of observing an evokes PSC of $\tilde{e}_i > 0$ in response to the ith AP is given by the mixture distribution

$$p(\tilde{e}_i|\theta) = \sum_{j=1}^{M} \binom{M}{j} (p_i p_r)^j \left(1 - p_i p_r\right)^{M-j} f(\tilde{e}_i, \langle c \rangle j, CV_q/\sqrt{j}) \tag{S86}$$

[98, 99]. This leads to the likelihood function for observing a sequence of evoked PSC $\tilde{e}_1, \tilde{e}_2, \ldots \tilde{e}_{N_{stimu}}$ for the first N_{stim} stimuli as

$$\ell(\theta) = \prod_{i=1}^{N_{stim}} p(\tilde{e}_i|\theta). \tag{S87}$$

Given data for the first $N_{stim} = 50$ stimuli from Fig 5A and assuming $CV_q = 20\%$, we maximized $\ell(\theta)$ using Microsoft Excel's Solver Toolbox with the GRC Nonlinear solving method. This procedure results in the following optimal sequence of refilling probabilities

$$p_{d,1} = 0.94,\ p_{d,2} = 0.74,\ p_{d,3} = 0.68,\ p_{d,4} = 0.55,\ p_{d,5} = 0.52 \ \&\ p_d = 0.52 \tag{S88}$$

that are similar to the values (25) obtained using least-square fitting. It is important to point out that given the high release probability, which results in almost uncorrelated successive evoked PSCs (Fig 5D), we have ignored correlations between $\tilde{e}_i$ in the likelihood formulation. In scenarios where significant correlation exists, using the framework proposed in [63] to formulate the likelihood function that explicitly takes into account $\tilde{e}_i$ correlations can dramatically improve synaptic quantal parameter estimates.

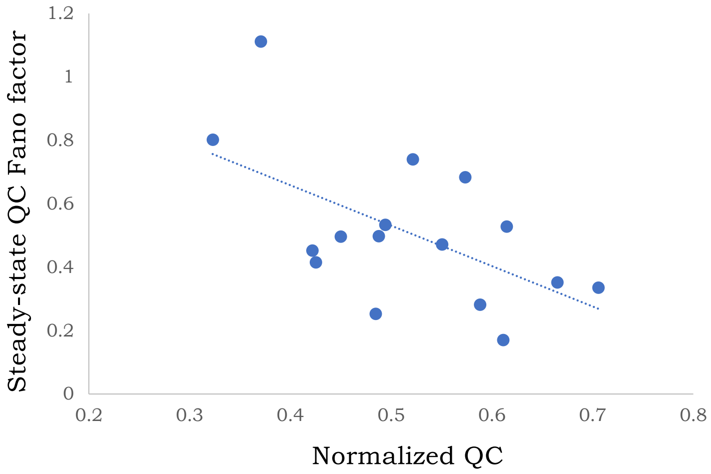

Fig C: The steady-state QC Fano factor as a function of the normalized QC (steady-state mean QC normalized to the first stimulus) for 14 MNTB-LSO connections from [69]. Each point represents the statistics evaluated from a single-cell QC recording at 50Hz stimulation using stimulus numbers 10 to 800. Consistent with (8), the steady-state QC Fano factor shows a negative correlation with normalized synaptic depression with a Pearson correlation coefficient of -0.6 ($R^2 = 0.38$), and a 95% confidence interval $[-0.85, -0.15]$.

Appendix G

Of the 19 single-cell recordings obtained from [69], we discarded 3 because of low QCs (QC for first stimulus less than 10). The steady-state QC Fano factor of the 16 single-cell recordings is shown in Fig C as a function of the normalized synaptic depression. To compute steady-state statistics (QC mean and FF) we used stimulus numbers 10 to 800 as some cells began to show a gradual decline in QC after that.

References

1. Schneggenburger R, Sakaba T, Neher E. Vesicle pools and short-term synaptic depression: lessons from a large synapse. Trends in Neurosciences. 2002;25(4):206–212.

2. Neher E. Some subtle lessons from the calyx of Held synapse. Biophysical Journal. 2017;112(2):215–223.

3. Elmqvist D, Quastel DM. A quantitative study of end-plate potentials in isolated human muscle. The Journal of Physiology. 1965;178(3):505–529.

4. Gotor JJR, Mahfooz K, Pérez-Otaño I, Wesseling JF. Parallel processing of quickly and slowly mobilized reserve vesicles in hippocampal synapses. eLife. 2024;12.

5. Alabi AA, Tsien RW. Synaptic vesicle pools and dynamics. Cold Spring Harbor Perspectives in Biology. 2012;4(8):a013680.

6. Tran V, Miki T, Marty A. Three small vesicular pools in sequence govern synaptic response dynamics during action potential trains. Proceedings of the National Academy of Sciences. 2022;119(5):e2114469119.

7. Datta P, Gilliam J, Thoreson WB, Janz R, Heidelberger R. Two pools of vesicles associated with synaptic ribbons are molecularly prepared for release. Biophysical Journal. 2017;113(10):2281–2298.

8. Durán E, Montes MÁ, Jemal I, Satterfield R, Young S, de Toledo GÁ. Synaptotagmin-7 controls the size of the reserve and resting pools of synaptic vesicles in hippocampal neurons. Cell Calcium. 2018;74:53–60.

9. Barros-Zulaica N, Rahmon J, Chindemi G, Perin R, Markram H, Muller E, et al. Estimating the readily-releasable vesicle pool size at synaptic connections in the neocortex. Frontiers in Synaptic Neuroscience. 2019;11:29.

10. Richards DA, Guatimosim C, Rizzoli SO, Betz WJ. Synaptic vesicle pools at the frog neuromuscular junction. Neuron. 2003;39(3):529–541.

11. Guarnieri FC. How do synaptic vesicles know which pool they belong to? Journal of Neuroscience. 2017;37(9):2276–2278.

12. Doussau F, Schmidt H, Dorgans K, Valera AM, Poulain B, Isope P. Frequency-dependent mobilization of heterogeneous pools of synaptic vesicles shapes presynaptic plasticity. eLife. 2017;6:e28935.

13. Mahfooz K, Singh M, Renden R, Wesseling JF. A well-defined readily releasable pool with fixed capacity for storing vesicles at calyx of Held. PLoS Computational Biology. 2016;12(4):e1004855.

14. Welzel O, Henkel AW, Stroebel AM, Jung J, Tischbirek CH, Ebert K, et al. Systematic heterogeneity of fractional vesicle pool sizes and release rates of hippocampal synapses. Biophysical Journal. 2011;100(3):593–601.

15. Wang LY, Kaczmarek LK. High-frequency firing helps replenish the readily releasable pool of synaptic vesicles. Nature. 1998;394(6691):384–388.

16. Zucker RS, Regehr WG. Short-term synaptic plasticity. Annual Review of Physiology. 2002;64:355–405.

17. Abbott LF, Varela J, Sen K, Nelson S. Synaptic depression and cortical gain control. Science. 1997;275(5297):221–224.

18. Hennig MH. Theoretical models of synaptic short term plasticity. Frontiers in Computational Neuroscience. 2013;7:45.

19. Taschenberger H, Scheuss V, Neher E. Release kinetics, quantal parameters and their modulation during short-term depression at a developing synapse in the rat CNS. The Journal of Physiology. 2005;568(2):513–537.

20. Le Bé J, Markram H. Spontaneous and evoked synaptic rewiring in the neonatal neocortex. Proceedings of the National Academy of Sciences of the United States of America. 2006;103:13214.

21. Wang Y, Markram H, Goodman PH, Berger TK, Ma J, Goldman-Rakic PS. Heterogeneity in the pyramidal network of the medial prefrontal cortex. Nature Neuroscience. 2006;9:534–542.

22. Tsodyks M, Markram H. The neural code between neocortical pyramidal neurons depends on neurotransmitter release probability. Proceedings of the National Academy of Sciences. 1997;94:719–723.

23. Abbott LF, Varela JA, Sen K, Nelson SB. Synaptic depression and cortical gain control. Science. 1997;275:221–224.

24. Tsodyks M, Pawelzik K, Markram H. Neural networks with dynamic synapses. Neural Computation. 1998;10:821–835.

25. Markram H, Wang Y, Tsodyks M. Differential signaling via the same axon of neocortical pyramidal neurons. Proceedings of the National Academy of Sciences. 1998;95:5323–5328.

26. Senn W, Markram H, Tsodyks M. An algorithm for modifying neurotransmitter release probability based on pre- and postsynaptic spike timing. Neural Computation. 2001;13:35–67.

27. Pulido C, Trigo FF, Llano I, Marty A. Vesicular release statistics and unitary postsynaptic current at single GABAergic synapses. Neuron. 2015;85:159–172.

28. Malagon G, Miki T, Llano I, Neher E, Marty A. Counting vesicular release events reveals binomial release statistics at single glutamatergic synapses. Journal of Neuroscience. 2016;36:4010–4025.

29. Faisal AA, Selen LPJ, Wolpert DM. Noise in the nervous system. Nature Reviews Neuroscience. 2008;9:292–303.

30. Chance FS, Nelson SB, Abbott LF. Synaptic depression and the temporal response characteristics of V1 cells. Journal of Neuroscience. 1998;18:4785–4799.

31. Rosenbaum R, Rubin JE, Doiron B. Short-term synaptic depression and stochastic vesicle dynamics reduce and shape neuronal correlations. Journal of Neurophysiology. 2013;109:475–484.

32. Schneidman E, Freedman B, Segev I. Ion channel stochasticity may be critical in determining the reliability and precision of spike timing. Neural Computation. 1998;10:1679–1703.

33. Zhang C, Peskin CS. Improved signaling as a result of randomness in synaptic vesicle release. Proceedings of the National Academy of Sciences. 2015;112:14954–14959.

34. Arleo A, Nieus T, Bezzi M, D'Errico A, D'Angelo E, Coenen OJMD. How synaptic release probability shapes neuronal transmission: information-theoretic analysis in a cerebellar granule cell. Neural Computation. 2010;22.

35. Robert P, Vignoud G. Stochastic models of neural synaptic plasticity. SIAM Journal on Applied Mathematics. 2021;81(5):1821–1846.

36. Manwani A, Koch C. Detecting and estimating signals over noisy and unreliable synapses: information-theoretic analysis. Neural Computation. 2001;13(1):1–33.

37. Rusakov DA, Savtchenko LP, Latham PE. Noisy synaptic conductance: bug or a feature? Trends in Neurosciences. 2020;43(6):363–372.

38. Kullmann DM. Quantal variability of excitatory transmission in the hippocampus: implications for the opening probability of fast glutamate-gated channels. Proceedings of the Royal Society of London Series B: Biological Sciences. 1993;253:107–116.

39. Goldman MS. Enhancement of information transmission efficiency by synaptic failures. Neural Computation. 2004;16:1137–1162.

40. Scheuss V, Neher E. Estimating synaptic parameters from mean, variance, and covariance in trains of synaptic responses. Biophysical Journal. 2001;81(4):1970–1989.

41. Scheuss V, Schneggenburger R, Neher E. Separation of presynaptic and postsynaptic contributions to depression by covariance analysis of successive EPSCs at the calyx of Held synapse. Journal of Neuroscience. 2002;22(3):728–739.

42. Bekkers JM. Quantal analysis of synaptic transmission in the central nervous system. Current Opinion in Neurobiology. 1994;4(3):360–365.

43. Lanore F, Silver RA. Extracting quantal properties of transmission at central synapses. Neuromethods. 2016;113:193–211.

44. Loebel A, Silberberg G, Helbig D, Markram H, Tsodyks M, Richardson MJE. Multiquantal release underlies the distribution of synaptic efficacies in the neocortex. Frontiers in Computational Neuroscience. 2009;3:27.

45. Silver RA, Momiyama A, Cull-Candy SG. Locus of frequency-dependent depression identified with multiple-probability fluctuation analysis at rat climbing fibre-Purkinje cell synapses. The Journal of Physiology. 1998;510(Pt 3):881–902.

46. Sargent PB, Saviane C, Nielsen TA, et al. Rapid vesicular release, quantal variability, and spillover contribute to the precision and reliability of transmission at a glomerular synapse. Journal of Neuroscience. 2005;25:8173–8187.

47. Lanore F, Labrousse VF, Szabo Z, et al. Deficits in Morphofunctional Maturation of Hippocampal Mossy Fiber Synapses in a Mouse Model of Intellectual Disability. Journal of Neuroscience. 2012;32:17882–17893.

48. Kobbersmed JR, Grasskamp AT, Jusyte M, Böhme MA, Ditlevsen S, Sørensen JB, et al. Rapid regulation of vesicle priming explains synaptic facilitation despite heterogeneous vesicle: Ca2+ channel distances. Elife. 2020;9:e51032.

49. Khedraki A, Reed EJ, Romer SH, Wang Q, Romine W, Rich MM, et al. Depressed synaptic transmission and reduced vesicle release sites in Huntington's disease neuromuscular junctions. Journal of Neuroscience. 2017;37(34):8077–8091.

50. Bykowska O, Gontier C, Sax AL, Jia DW, Montero ML, Bird AD, et al. Model-based inference of synaptic transmission. Frontiers in Synaptic Neuroscience. 2019;11.

51. Vahdat Z, Xu Z, Singh A. Modeling and characterization of neuronal synapses using stochastic hybrid systems. In: 2019 IEEE 58th Conference on Decision and Control (CDC). IEEE; 2019. p. 4729–4734.

52. Singh A. Noise mechanisms in synaptic transmission and their impact on spike-timing precision. In: 2017 IEEE 56th Annual Conference on Decision and Control (CDC). IEEE; 2017. p. 5925–5930.

53. Vahdat Z, Singh A. Frequency-dependent modulation of stochasticity in postsynaptic neuron firing times. In: 2022 IEEE 61st Conference on Decision and Control (CDC); 2022. p. 635–640.

54. Elmqvist D, Quastel D. A quantitative study of end-plate potentials in isolated human muscle. The Journal of Physiology. 1965;178(3):505.

55. Kusick GF, Ogunmowo TH, Watanabe S. Transient docking of synaptic vesicles: implications and mechanisms. Current Opinion in Neurobiology. 2022;74:102535.

56. Kusick GF, Chin M, Raychaudhuri S, Lippmann K, Adula KP, Hujber EJ, et al. Synaptic vesicles transiently dock to refill release sites. Nature Neuroscience. 2020;23(11):1329–1338.

57. Tsodyks MV, Markram H. The neural code between neocortical pyramidal neurons depends on neurotransmitter release probability. Proceedings of the National Academy of Sciences. 1997;94(2):719–723.

58. Barri A, Wang Y, Hansel D, Mongillo G. Quantifying repetitive transmission at chemical synapses: a generative-model approach. eNeuro. 2016;3:0113–15.2016. 113–115.

59. Fuhrmann G, Segev I, Markram H, Tsodyks M. Coding of temporal information by activity-dependent synapses. Journal of Neurophysiology. 2002;87(1):140–148.

60. De La Rocha J, Parga N. Short-term synaptic depression causes a non-monotonic response to correlated stimuli. Journal of Neuroscience. 2005;25(37):8416–8431.

61. Zhang C, Peskin CS. Analysis, simulation, and optimization of stochastic vesicle dynamics in synaptic transmission. Communications on Pure and Applied Mathematics. 2020;73(1):3–62.

62. Rijal K, Müller NI, Friauf E, Singh A, Prasad A, Das D. Exact distribution of the quantal content in synaptic transmission. Physical Review Letters. 2024;132(22):228401.

63. Bird AD, Wall MJ, Richardson MJE. Bayesian inference of synaptic quantal parameters from correlated vesicle release. Frontiers in Computational Neuroscience. 2016;10.

64. Krächan EG, Fischer AU, Franke J, Friauf E. Synaptic reliability and temporal precision are achieved via high quantal content and effective replenishment: auditory brainstem versus hippocampus. The Journal of Physiology. 2017;595(3):839–864.

65. Lujan BJ, Singh M, Singh A, Renden RB. Developmental shift to mitochondrial respiration for energetic support of sustained transmission during maturation at the calyx of Held. Journal of Neurophysiology. 2021;126(4):976–996.

66. Lujan B, von Gersdorff H. Tuning auditory synapses for resilience, reliability and precision. The Journal of Physiology. 2017;595(3):621.

67. Müller NIC, Paulußen I, Hofmann LN, Fisch JO, Singh A, Friauf E. Development of synaptic fidelity and action potential robustness at an inhibitory sound localization circuit: effects of otoferlin-related deafness. The Journal of Physiology. 2022;600(10):2461–2497.

68. Brill SE, Maraslioglu A, Kurz C, Kramer F, Fuhr MF, Singh A, et al. Glycinergic transmission in the presence and absence of functional GlyT2: lessons from the auditory brainstem. Frontiers in Synaptic Neuroscience. 2021;12:56.

69. Brill SE, Janz K, Singh A, Friauf E. Considerable differences between auditory medulla, auditory midbrain, and hippocampal synapses during sustained high-frequency stimulation: exceptional vesicle replenishment restricted to sound localization circuit. Hearing Research. 2019;381:107771.

70. Friauf E, Krchan EG, Müller NIC. Lateral superior olive: organization, development, and plasticity. In: The Oxford Handbook of the Auditory Brainstem. Oxford University Press; 2019.

71. Ashida G, Tollin DJ, Kretzberg J. Physiological models of the lateral superior olive. PLOS Computational Biology. 2017;13(12):e1005903.

72. Franken TP, Joris PX, Smith PH. Principal cells of the brainstems interaural sound level detector are temporal differentiators rather than integrators. eLife. 2018;7:e33854.

73. Grothe B, Pecka M, McAlpine D. Mechanisms of sound localization in mammals. Physiological Reviews. 2010;90(3):983–1012.

74. Gillespie DC, Kim G, Kandler K. Inhibitory synapses in the developing auditory system are glutamatergic. Nature Neuroscience. 2005;8(3):332–338.

75. Rosenbaum R, Rubin J, Doiron B. Short term synaptic depression imposes a frequency dependent filter on synaptic information transfer. PLOS Computational Biology. 2012;8:e1002557.

76. Bird AD, Richardson MJE. Transmission of temporally correlated spike trains through synapses with short-term depression. PLOS Computational Biology. 2018;14(6):1–25.

77. Zhang Y, Li D, Darwish Y, Fu X, Trussell LO, Huang H. KCNQ channels enable reliable presynaptic spiking and synaptic transmission at high frequency. Journal of Neuroscience. 2022;42(16):3305–3315.

78. Fuhrmann G, Cowan A, Segev I, Tsodyks M, Stricker C. Multiple mechanisms govern the dynamics of depression at neocortical synapses of young rats. The Journal of Physiology. 2004;560(Pt 2):423–431.

79. Costa RP, Sjöström PJ, van Rossum MCW. Probabilistic inference of short-term synaptic plasticity in neocortical microcircuits. Frontiers in Computational Neuroscience. 2013;7.

80. Dorgans K, Demais V, Bailly Y, Poulain B, Isope P, Doussau F. Short-term plasticity at cerebellar granule cell to molecular layer interneuron synapses expands information processing. eLife. 2019;8:e41586.

81. Weichard I, Taschenberger H, Gsell F, Bornschein G, Ritzau-Jost A, Schmidt H, et al. Fully-primed slowly-recovering vesicles mediate presynaptic LTP at neocortical neurons. Proceedings of the National Academy of Sciences. 2023;120(43):e2305460120.

82. Lin KH, Taschenberger H, Neher E. A sequential two-step priming scheme reproduces diversity in synaptic strength and short-term plasticity. Proceedings of the National Academy of Sciences. 2022;119(34):e2207987119.

83. Silva M, Tran V, Marty A. A maximum of two readily releasable vesicles per docking site at a cerebellar single active zone synapse. eLife. 2024;12:RP91087.

84. Rizzoli SO. Synaptic vesicle recycling: steps and principles. The EMBO Journal. 2014;33(8):788–822.

85. Gambrell O, Singh A. Consequences of decoy site repair and feedback regulation on neurotransmission dynamics. bioRxiv. 2024; p. 2024–07.

86. Ford CP. The role of D2-autoreceptors in regulating dopamine neuron activity and transmission. Neuroscience. 2014;282:13–22.

87. Calipari ES, Sun H, Eldeeb K, Luessen DJ, Feng X, Howlett AC, et al. Amphetamine self-administration attenuates dopamine D2 autoreceptor function. Neuropsychopharmacology. 2014;39(8):1833–1842.

88. Formisano R, Mersha MD, Caplan J, Singh A, Rankin CH, Tavernarakis N, et al. Synaptic vesicle fusion is modulated through feedback inhibition by dopamine auto-receptors. Synapse. 2020;74(1):e22131.

89. Formisano R, Rosikon KD, Singh A, Dhillon HS. The dopamine membrane transporter plays an active modulatory role in synaptic dopamine homeostasis. Journal of Neuroscience Research. 2022;100(8):1551–1559.

90. Vahdat Z, Gambrell O, Singh A. Negative feedback regulation via an autapse enhances neuronal firing precision. In: 2024 American Control Conference (ACC). IEEE; 2024. p. 3941–3946.

91. Binda F, Spaeth L, Kumar A, Isope P. Excitation and inhibition delays within a feedforward inhibitory pathway modulate cerebellar purkinje cell output in mice. Journal of Neuroscience. 2023;43(33):5905–5917.

92. Gambrell O, Vahdat Z, Singh A. Feedforward regulation of interneuronal communication. In: 2024 IEEE 63rd Conference on Decision and Control (CDC). IEEE; 2024. p. 607–612.

93. Singh A. An exact transient solution characterizing stochastic dynamics of neuronal synaptic transmission. OSF Preprints. 2023;.

94. Soltani M, Singh A. Moment-based analysis of stochastic hybrid systems with renewal transitions. Automatica. 2017;84:62–69.

95. Singh A, Hespanha JP. Stochastic hybrid systems for studying biochemical processes. Philosophical Transactions of the Royal Society A. 2010;368:4995–5011.

96. Soltani M, Singh A. Moment analysis of linear time-varying dynamical systems with renewal transitions. SIAM Journal on Control and Optimization. 2019;57(4):2660–2685.

97. Gambrell O, Vahdat Z, Singh A. Feedback and feedforward regulation of interneuronal communication. bioRxiv. 2024; p. 2024–03.

98. Bhumbra GS, Beato M. Reliable evaluation of the quantal determinants of synaptic efficacy using Bayesian analysis. Journal of Neurophysiology. 2013;109:603–620.

99. Turner DA, West M. Bayesian analysis of mixtures applied to post-synaptic potential fluctuations. Journal of Neuroscience Methods. 1993;47(1-2):1–21.
