# Supplementary material for: Inferring synaptic transmission from the stochastic dynamics of the quantal content: An analytical approach
Source: PLoS Comput Biol. 2025 May 13;21(5):e1013067. doi: 10.1371/journal.pcbi.1013067 (PMC12101786; doi:10.1371/journal.pcbi.1013067)
Supplement: Attachment — Submitted filename: response.pdf [file pcbi.1013067.s003.pdf]

Response to comments from Referee #1

The authors derive a number of analytical results on the distribution of synaptic vesicles before and during vesicle release. They apply their results to published data on auditory synapses, which have several interesting properties due to their need for high-speed transmission. They find that the data is inconsistent with a constant rate of vesicle replenishment in this system.

Response: We thank the reviewer for the valuable suggestions and for appreciating our work. We have revised the paper to put our results in the context of the literature and have cited all of the work suggested by the referee. Apart from the changes made in the main text (highlighted in red color), we would particularly like to highlight three major changes in the Appendices in the Supplementary Information:

- We expanded Appendix A to consider refilling and undocking occurring as per inhomogeneous Poisson processes, where these rates are not constant but vary with the timing of the last AP.

- We have added a new Appendix E to consider quantal size fluctuations, and how they impact the Fano factor and Pearson correlation coefficient of evoked PSCs. These results have led to a new Fig. S2 where the robustness of parameter estimates to quantal size fluctuations is explored.

- Another new Appendix F has been added to the manuscript, where we have used a likelihood-based formulation using mixture-model distributions that consider fluctuations in the quantal size to infer the time-varying refilling rates.

The major weakness of this study is that its results are not placed in the context of existing results and this makes it very hard to disentangle the novel contributions that are made by the paper. For example: The fact that fluctuations are necessary to identify quantal parameters has been known since (at least) Bekkers (Curr Op Neurobiol, 1994) and has a well-known solution in mean-variance analysis (Silver, Momiyama, Cull-Candy, J Physiol 1998). Loebel, Silberberg et al (Front Comp Neuro, 2009) provides a nice example and the method is reviewed in Lanore and Silver (Neurometh, 2016). In this paper, none of this work is acknowledged and a very similar solution is introduced as if it were entirely novel (see for example italic emphasis at the end of section III).

Response: We have now added a new paragraph providing a brief introduction to

mean-variance analysis and more recent likelihood methods. More specifically we have added (lines 19-32):

There is a rich tradition of using QC fluctuation statistics arising from the inherent stochastic nature of transmitter releases to infer synaptic parameters [42]. The classical approach for doing this is the variance-mean analysis, where the parabolic relationship between the variance and mean amplitudes of postsynaptic responses is fitted to corresponding statistics obtained from data by varying the release probability [43]-[45]. The variance-mean analysis has been applied to infer quantal parameters at diverse central synapses [45]-[47], and the neuromuscular junction [48],[49]. A key assumption of this approach is that synaptic responses remain temporally stable for sufficiently long durations to give reliable estimates of the mean and variance. Building on this tradition of leveraging fluctuations, several recent methods generalize the approach to consider short-term plasticity by finding parameters that maximize the likelihood of observing a sequence postsynaptic responses to a train of APs given an underlying stochastic model of transmitter release [50].

The emphasized sentence has also been revised to (lines 164-167):

Consistent with previous work [43],[50], these hypothetical examples emphasize that QC noise statistics contain useful signatures providing additional insights into the mechanisms underlying synaptic depression.

> *The introduction of time-varying parameters for vesicle release and recovery is interesting. Some mechanisms giving rise to this are discussed in Fuhrmann et al (J Physiol, 2004). However, the analytical results presented in the paper drop this assumption (either assuming time independence or steady-state behaviour), which means that the majority of equations in the Results appear to be special cases of results already derived in Goldman (Neural Comp, 2004), Rosenbaum, Rubin, and Doiron (PLoS CB, 2012) (both of which the manuscript cites), and in Bird and Richardson (PLoS CB, 2018) (which the manuscript doesnÕt). It would be interesting to see exactly where this study has been able to identify new and significant results.*

Response: We have rewritten lines both in the Introduction and the Discussion to reemphasize the novelty of the theoretical work. For example, in Introduction, we write (lines 45-48):

The key analytical contribution of this paper is the *exact* analytical solution for

the transient statistical distribution of the quantal content (QC) for such a general stochastic model of synaptic transmission with time-varying parameters.

We now leverage this analytical solution for a stimulus-dependent refilling probability to define a likelihood function in the newly-addedAppendix F, that is then optimized to infer the varying refilling probabilities that are key to understanding short-term plasticity at the MNTB-LSO synapses. With respect to our results on steady-state statistics we have added the following paragraph in the Discussion (lines 310-323):

By further investigating the process during steady-state transmission, we derive exact formulas for the steady-state QC distribution, its associated Fano factor FF, the correlation coefficient ρ between successive QCs, and QC autocorrelation function as a function of the steady-state refilling, undocking and release probabilities p_d, p_u and p_r, respectively. In Appendix E and F these formulas are extended to consider quantal size fluctuations. Such steady-state statistics for synaptic transmissions have also been previously reported in the case of no undocking $p_u = 0$, and docking site refilling occurring as per a memoryless Poisson process [62],[75],[76]. As highlighted in Appendix A, the proposed modeling framework also relaxes this assumption by allowing the refilling/undocking rates in the interstimulus interval to vary arbitrarily with precise AP timing introducing some form of memory in these processes. As we point out later, relaxation of this memoryless assumption is key to understanding synaptic transmission with random inter-stimulus times.

> *The analysis of experimental data is also unsatisfying. The comparison to existing methods for the data analysis (above Fig 6) uses Elmqvist and Quastel (J Physiol, 1965), a paper almost 60 years old and does not reference any more modern methods..*

Response: Our purpose of invoking the EQ method was simply to illustrate the point that the assumption employed for applying this method (no vesicle replenishment during the first 50 ms) is violated in this case leading to much lower release probabilities that are inconsistent with the evoked PSCs. We have now rewritten this part of the manuscript (please see around Eq. 24) to emphasize this point (lines 230-244).

Towards the end of the section, we added new material on the analysis of short-

term plasticity seen in the experimental data using both least-square fitting and a likelihood-based estimation approach that accounts for quantal size fluctuations. More specifically, we have added (lines 262-275):

Since the release probability is already quite high and close to one at steady-state (Fig. 6C), we considered a model with a time-varying refilling probability to explain the observed short-term plasticity. More specifically, we considered different refilling probabilities $p_{d,1}$,$p_{d,2}$, $p_{d,3}$, $p_{d,4}$,$p_{d,5}$ for the first five stimuli, and then a fixed probability $p_d = p_{d,i}, \forall i \geq 6$ for the remaining stimuli. Recall that our proposed analytical framework provides an exact statistical QC distribution at each stimulus for such time-varying refilling probabilities. To infer these parameters we consider two alternative approaches. The first approach performs a least-square fit between the model-predicted mean QC and data, and this results in

$$p_{d,1} = 0.92,\ p_{d,2} = 0.73,\ p_{d,3} = 0.66,\ p_{d,4} = 0.53,\ p_{d,5} = 0.12\ \&\ p_d = 0.51 \qquad (1)$$

i.e., a refilling probability that starts high and then reaches its steady-state value within the first six stimuli (inset of Fig. 6B plots the inferred refilling probabilities). In the alternative approach, we infer the parameter using a maximum likelihood approach that explicitly takes into account quantal size fluctuations and results in parameter values similar to (1) (see Appendix F for details).

> *Further the authors neglect another potential source of variability in synaptic responses: variability in the response to a single vesicle. This has been acknowledged as a major problem since Kuno (J Physiol, 1964). Studies such as Turner and West (J Neurosci Meth, 1993) and Bhumbra and Beato (J Neurophysiol, 2013) have already applied mixture models to disentangle the effects of vesicle variability and quantal stochasticity for single pulses.*

Response: This is an excellent suggestion and we had completely ignored quantal size fluctuations earlier. In response, we have now added a new Appendix E and Fig. S2 to explore this effect in detail. Analytically, we first extended the formulas for the Fano factor and the Pearson correlation coefficient to account for quantal size fluctuations and then explored its impact on parameter estimation. We quantify the magnitude of these fluctuations using the coefficient of variation of quantal size CV_q. Concerning the parameter estimation for the steady-state refilling and release probabilities for the MNTB-LSO synapse, we have added in Appendix E:

To study the impact of quantal size fluctuations, we solve these equations numerically for increasing values of CV_q^2. The results are shown in Fig. S2 where with increasing CV_q, p_r shows a slight decrease (from 0.93 to 0.91) and p_d shows an increase (from 0.53 to 0.70) as CV_q increases from 0 to 40%. Quantal size data from individual MNTB-LSO synaptic connections reveal fluctuations of magnitude $CV_q \approx 10 - 20\%$ (data not shown). Based on our results in Fig. S2, we don't expect significant changes in the inferred parameter values in this range.

In Appendix F, we formulate a mixture model to capture the distribution for the evoked PSCs considering gamma-distributed quantal sizes and use that for a likelihood estimation of the dynamically changing refilling rate during the initial train of APs

> *The above methods have also been extended by Barri, Wang et al (eNeuro, 2016) and Bird, Wall, and Richardson (Front Comp Neuro, 2016) to account for synaptic plasticity. Both papers also explicitly leverage the serial correlations between quantal events to improve their estimates of the data, something that is again presented as a novelty here. It would be very interesting to see how the analysis conducted here compares to the more recently published literature. Bykowska, Gontier et al (Front Syn Neuro, 2019) provide a more recent review of methods that have been used to solve this problem..*

Response: We sincerely thank the reviewer for bringing this work to our attention and we have now cited all these works. We have changed the language in the manuscript (see above Eq. 19 and below Eq. 113) emphasizing that leveraging ePSC correlations has been known to improve synaptic quantal parameter estimates in the literature. Indeed our experimental observation of uncorrelated QCs in Fig. 5D was the key to inferring the steady-state refilling and release probabilite together with the observed statistical dispersion in QCs. We also added the following statement in the Discussion (lines 340-348):

It is important to emphasize that knowing the mean QC and FF are by itself not sufficient to infer steady-state quantal parameters and knowledge of correlations is necessary to be able to uniquely infer p_r and p_d. The approach of solving statistical lower-order moments (16) and (20) to infer p_r and p_d is analytically tractable and can be extended to consider quantal size fluctuations (Appendix E). The parameters determined from here can be used in conjunction with computationally expensive

likelihood-based approaches for parameter inference that explicitly take into account correlations between successive evoked PSCs .

> *The general focus on fixed interspike intervals (periodic spike trains) is a weakness if the method is to be used for inference as Costa, Sjostrom, and van Rossum (Front Comp Neuro, 2013) showed that variable (in particular uncorrelated exponential) interspike intervals are optimal for inferring the parameters of short-term plasticity.*

Response: This is a good point and related to some of our future work on gamma-distributed interspike intervals where preliminary data points to an inhomogenous Poisson rate of refilling deviating from the classical assumption of a fixed Poisson rate of refilling. We have added this paragraph in the Discussion (lines 383-392):

While in this manuscript we analyzed data from [69] based on a fixed inter-stimulus interval, it has been shown that variable inter-stimulus interval (more specifically, a Poisson AP train) can provide more accurate parameter estimates [79]. In future work, we want to consider gamma-distributed inter-stimulus intervals where the noise in the intervals can be arbitrarily modulated. Preliminary data using this protocol on the MNTB-LSO synapse reveals SV refilling at docking sites occurring with rates that depend on the precise timing of the last AP, and this phenomenon can be incorporated into our general stochastic modeling framework with refilling occurring as per an inhomogeneous Poisson process (Appendix A).

> *The finding that a time-varying replacement probability is necessary to fit the LSO data is interesting. Such a mechanism appears similar to the frequency-dependent recovery model introduced by Fuhrmann et al (J Physiol, 2004) and implemented as the FDR model in Bird, Wall, and Richardson (Front Comp Neuro, 2016). It would be particularly interesting if the authors could discuss this model and identify if time or frequency dependence is a more significant factor in vesicle recovery at these synapses.*

Response: This is an interesting point. We discuss the similarities in the Discussion where we have added (lines 371-379):

Finally, analysis of the transient depression in QCs seen in data (Fig. 5B) indicates that the short-term plasticity is a result of a decreasing refilling probability (inset in Fig. 6B). We plan to capture this phenomenon more mechanistically in future work

by considering an SV pool that feeds into the readily releasable pool. It is interesting to note similarities between this model and the release-independent depression reported in [78] that is mediated by a decrease in the release probability to subsequent APs. However, it is important to note the key difference with the MNTB-LSO synapse where the decrease occurs in the rate of refilling of empty docking sites with SVs .

Previous work on MNTB-LSO synapses has looked at recovery after sustained stimulation with various frequencies (1 min, 1-200 Hz; for example, see Fig. 2Ac in Krachan et al, Synaptic reliability and temporal precision are achieved via high quantal content and effective replenishment: auditory brainstem versus hippocampus. Journal of Physiology, 2016. https://pubmed.ncbi.nlm.nih.gov/27673320/) The figure shows recovery from depression after stimulation at eight frequencies. Here the steady-state depression in QC is lower when stimulation frequencies are higher, opposite to the FDR phenomenon.

> *Overall, although the authors have done good and careful work in this manuscript, the way it is written makes it very hard to identify a novel contribution to the field. If the manuscript could be extensively rewritten in the context of the literature mentioned above (and any other important papers I have missed) so that similarities with, differences from, and improvements over existing work are clearer, it would be much easier to assess the significance of the findings.*

Response: We are again very grateful for the reviewer's suggestions that have greatly improved the quality of the manuscript and highlighted its novel contributions (both theoretical and for the auditory MNTB-LSO synapse) to the existing literature.

Response to comments from Referee #2

> *Understanding the dynamics of neurotransmitter filled synaptic vesicles (SVs) is important to understand interactions between neurons. These vesicles are docked at sites in the axon terminal and are released when an action potential (Aps) arrives at the axon terminal. The depletion of these SVs due to successive APs is counteracted by replenishment. These processes (release and replenishment/docking) are inherently probabilistic. In this manuscript authors have built a probabilistic model of SV docking and release. They analytically derive the probability distribution of both occupation of sites and quantal content (QC), and show that it is binomial. They also derive formulas for fano factor (FF) and QC correlations (ρ). They show that different parameter regimes that produce the same average behaviour can be distinguished based FF and ρ. They apply their results to electrophysiological data obtained from 50 Hz stimulation of MNTB-LSO synapse. They show that the method of Elmqvist and Quastel explains the mean synaptic depression but not the fluctuations in QC. They combine information from average synaptic depression, FF, and ρ to infer model parameters that better explain the fluctuation statistics of the QC. Overall this is a very interesting paper which not only provides a biologically interpretable model of vesicle docking and release but authors also provide an easy scheme to infer model parameters from experimental data. In principle I support the publication of the paper. There are only a few points where I think authors need to provide more clarification or possibly extend their model*

Response: We thank the reviewer for elegantly summarizing the work and providing valuable suggestions that we have now addressed.

> *Authors have only studied synaptic depression. How the results would be for synaptic facilitation. Authors should include at least one example of parameter setting that would lead to synaptic facilitation*

Response: This is a very good suggestion. We have now modified Fig. 2 and its caption to consider a fourth case of a time-varying release probability that results in synaptic facilitation.

> *The equations are symmetric for p_d and p_r. Is there a reason for considering only the former to vary with time and not the latter?*

Response: We now explicitly mention the reason at the end of page 11 (lines 262-275). Since $p_r \approx 0.93$ at steady-state is already very high, starting with a value of $p_{r,1} = 1$ (release probability for the first stimulus) will not explain the reduction

in QC by 50% at steady-state (compared to the first stimulus) as seen in the data (Figs. 5 A & B). We also point out that we have now added two new appendices for studying how quantal size fluctuations impact parameter estimates (Appendix E), and a likelihood-based approach for inferring the transient reduction in refilling probability (Appendix F).

> *The authors do not comment on the frequency dependence of synaptic plasticity in their model. In appendix A they relate the probabilities to rate kinetics and time period of stimulus, but do not mention how these rate kinetics themselves depend on frequency of the input. I think this is an important issue that needs to be elaborated.*

Response: We agree on this and now explicitly mention this point in Appendix A. We have also expanded this Appendix to consider refilling and undocking processes occurring as per inhomogeneous Poisson processes, where for example, the instantaneous refilling rate depends on the time elapsed since the last AP.
